# Changes in Concentration of Selected Biomarkers of Exposure in Users of Classic Cigarettes, E-Cigarettes, and Heated Tobacco Products—A Narrative Review

**DOI:** 10.3390/ijms26051796

**Published:** 2025-02-20

**Authors:** Justyna Śniadach, Aleksandra Kicman, Anna Michalska-Falkowska, Kamila Jończyk, Napoleon Waszkiewicz

**Affiliations:** 1 Department of Psychiatry, The Faculty of Medicine, Medical University of Bialystok, 15-272 Bialystok, Poland; k.jonczyk9606@gmail.com; 2 Department of Aesthetic Medicine, The Faculty of Pharmacy, Medical University of Bialystok, 15-267 Bialystok, Poland; olakicman@gmail.com; 3 MUB Biobank Data Repository, Medical University of Bialystok, 15-269 Bialystok, Poland; anna.michalska-falkowska@umb.edu.pl

**Keywords:** classic cigarettes, e-cigarettes, heated tobacco products, biomarkers, pro-inflammatory cytokines, anti-inflammatory cytokines, growth factors, matrix metalloproteinase 9, C-reactive protein, parameters of oxidative stress, microplastic, reduction programmes

## Abstract

Currently, the number of e-cigarette and heated tobacco product (HTP) users are steadily increasing, while the number of classic cigarette users are decreasing. The effects of smoking classic cigarettes on human health have been thoroughly described in the literature, but the negative health effects of e-cigarettes and HTPs on the human body are not clearly defined. Among users of different forms of tobacco, those at a particularly high risk of developing particular disease entities should be identified, allowing for the faster implementation of potential treatments, including psychotherapeutic ones. Biomarkers are used for this purpose. This paper summarizes the potential of these compounds from the different exposure groups of classic cigarettes, e-cigarettes, and HTPs, and presents changes in their concentrations in the body fluids of different tobacco users. This review discusses the impact of tobacco use in relation to levels of the following biomarkers: TNF-α, IL-1β, IL-6, IL-8, IL-17, IFN-γ, IL-10, IL-4, Il-13, TGF-β, VEGF EGF, HGF, BDNF, MMP-9, CRP, microplastics, and selected parameters of oxidative stress. This review also includes suggested forms of treatment, including Tobacco Product Use Reduction Programs, to minimize the potential negative effects of the above-mentioned products.

## 1. Introduction

Currently, about 1.3 billion people in the world smoke cigarettes. An estimated 6 billion cigarettes are smoked each year. About 80% of smokers live in developing countries, of which about 360 million live in China [1]. In Poland, about 9 million people smoke [2]. Currently, the number of deaths from tobacco-related diseases worldwide exceeds 8 million per year, of which nearly 1.3 million are passive smokers [3,4]. According to the World Health Organization, if the current trend continues, 8 million people could die each year from smoking in 2030 [5,6,7]. The predominant use of tobacco products is cigarettes, although there has been a decline in their use; at the same time, there has been a significant increase in the number of people using electronic cigarettes and heated tobacco products [8,9,10]. However, regardless of the type of tobacco used, smoking is associated with the development of a number of diseases such as cardiovascular [11] and respiratory [12] diseases and cancer [13].

There is not the slightest doubt that smoking addiction is a serious health problem that has been classified among the major addiction disorders. In the Diagnostic and Statistical Manual of Mental Disorders DSM-5 (American Psychiatric Association, 2013, 5th edition and subsequent revisions), Tobacco Use Disorder is classified as a problematic pattern of tobacco use leading to a clinically significant disorder or discomfort [14]. From this perspective, it is important to present the epidemiological and neurobiological aspects of this disorder.

Nearly 80% of tobacco smokers begin their habit in childhood or adolescence. Risk factors for smoking in childhood or adolescence include parental and peer influence, behavioral disorders, personality traits, and genetic factors [15]. It should be mentioned that the prevalence of nicotinism is markedly elevated in the population of people with mental illness. This is related to a common genetic background, the ability of nicotine to alleviate some mental disorders and the inhibitory effect of tobacco smoke on the activity of monoamine oxidases [16,17,18].

The largest group of cigarette addicts are those in the 20–39 age range—about 35% of the total smoking population. Cigarette smoking is more prevalent in people with secondary or vocational education than in those with higher education. In addition, marital status, having offspring, occupation, employment relationship, job stress, and earnings can be considered as factors influencing cigarette smoking [19]. Women are more likely to use cigarettes in response to conditioned stimuli and negative emotions, while men are more likely to use cigarettes in response to pharmacological stimuli. Contributing to this is the fact that men tend to metabolize nicotine more slowly than women, making them less likely to reach for cigarettes to maintain adequate nicotine levels throughout the day (Perkins et al., 2002 [19]; Perkins and Scott, 2008 [20]).

Smoking addiction depends on the positive reinforcement and hedonic effects of nicotine, but also on escaping the aversive consequences of nicotine withdrawal. Many studies suggest that avoidance of negative emotions accompanying withdrawal is a motivational element that promotes continued smoking and relapse after cessation. Most smokers (~70%) say they are interested in quitting, and nearly 55% attempt to quit within a year. However, only 7% of smokers are in abstinence after 1 month of quitting, and less than 2% do not return to cigarette smoking after 1 year [21].

Due to the growing number of smokers of e-cigarettes and heated tobacco products, it is advisable to isolate from the group of smokers those who are potentially at a higher risk of developing tobacco-related diseases. For this purpose, the determination of various types of biomarkers of exposure—substances that potentially indicate individuals with higher exposure to the occurrence of the aforementioned diseases—can be used [22,23]. Such compounds can be pro- or anti-inflammatory mediators. This paper summarizes the existing knowledge on changes in the concentrations of selected pro- and anti-inflammatory mediators and growth factors in the body fluids of various tobacco users and their potential use as biomarkers of exposure [24].

## 2. Background

**Figure 1:**
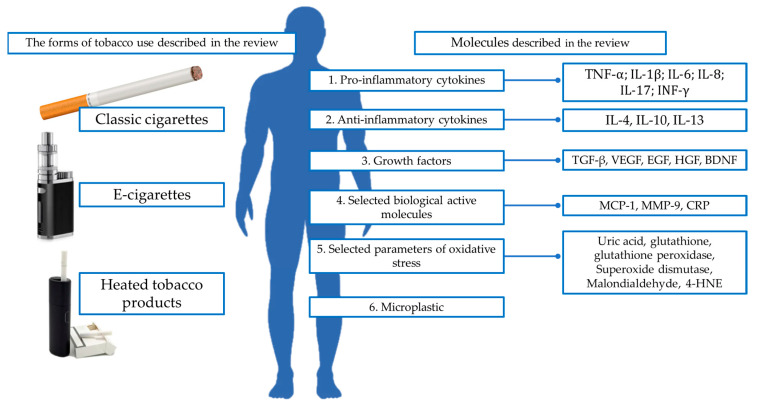
Tobacco use forms and molecule groups selected for this paper.

The figure below shows the most important biologically active compounds in nicotine users that are best described in the literature in three major forms—classic cigarettes, e-cigarettes, and heated tobacco products. The compounds were divided into the following six groups: (1) pro-inflammatory cytokines, (2) anti-inflammatory cytokines, (3) growth factors, (4) selected biological active parameters, (5) selected oxidative stress parameters, and (6) microplastics. This is shown in Figure 1.

The selection of indicators for analysis in scientific research is crucial for obtaining a complete picture of the impact of various factors on human health. For the present study, the described compounds were selected after a thorough analysis of the literature, which is described in the methodology section. It is worth mentioning here that in the case of studies on the effects of tobacco on the body, the choice of indicators such as pro-inflammatory and anti-inflammatory cytokines, growth factors, selected biological active molecules, selected parameters of oxidative stress, and microplastics is essential for obtaining accurate and comprehensive results.

Pro-inflammatory and anti-inflammatory cytokines are key mediators of the immune and inflammatory response. Selecting indicators such as TNF-α, IL-1β, IL-6, IL-8, IL-17, IFN-γ, IL-4, IL-10, and IL-13 allows us to assess the balance between pro-inflammatory and anti-inflammatory processes in the body. Understanding how different forms of tobacco affect the concentrations of these cytokines can help identify the mechanisms leading to inflammatory and autoimmune diseases [2,8,12].

Growth factors such as TGF-β, VEGF, EGF, HGF, and BDNF play key roles in regenerative processes, angiogenesis, and neuroplasticity. The analysis of the concentrations of these factors makes it possible to assess the impact of tobacco on repair and adaptive processes in the body. An increase in the concentrations of these factors may indicate an attempt to compensate for the damage caused by tobacco smoke [9,11,13].

Biologically active molecules such as MCP-1, MMP-9, and CRP are important markers of inflammation and tissue degradation. The analysis of these molecules allows for the assessment of the extent of tobacco-induced damage and potential mechanisms leading to chronic inflammatory and degenerative diseases [10,22,23].

Oxidative stress plays a key role in the pathogenesis of many chronic diseases, including cardiovascular disease, cancer, and neurodegenerative diseases. A selection of indicators such as uric acid, glutathione, glutathione peroxidase, superoxide dismutase, malondialdehyde, and 4-hydroxynonenal allows for the assessment of the degree of tobacco-induced oxidative stress and its potential health effects [1,3,7].

The presence of microplastics in the bodies of tobacco users is a new and important area of research. High concentrations of microplastics correlate with elevated levels of inflammatory biomarkers, suggesting that microplastics may contribute to chronic inflammation. The analysis of microplastics allows for the assessment of additional health risks associated with tobacco use [4,5,6].

In conclusion, the choice of these indicators for analysis is justified by their key role in inflammatory, regenerative, adaptive, and oxidative processes. This allows for a more complete picture of the impact of various forms of tobacco on human health and the identification of potential mechanisms leading to chronic diseases. The results of these studies may contribute to a better understanding of the health risks associated with tobacco use and to the development of more effective preventive and therapeutic strategies [24,25,26].

### The Role of Nicotine in the Addiction Process and Its Effects on Cholinergic Receptors

Nicotine, the main active ingredient in tobacco, plays a key role in the addiction process through its action on nicotinic cholinergic receptors in the mesolimbic centers of the brain. These receptors are involved in the regulation of monoamine, neurotransmitters, and neuropeptide systems, which has important implications for brain function and mental well-being. The effect of nicotine on dopamine secretion in the mesolimbic system leads to feelings of euphoria, but also to the development of addiction. Dopamine plays a key role in the brain’s reward system, and its elevated concentration induced by nicotine is responsible for the positive reinforcement associated with smoking [27].

In addition to its effects on the dopaminergic system, nicotine also affects the serotonergic and noradrenergic systems, which can lead to increased levels of anxiety and depression in addicts. Serotonin and norepinephrine are key mood-regulating neurotransmitters, and their disruption can have a significant impact on mental health. Studies have shown that long-term nicotine use can lead to a reduction in the number of nicotinic receptors, which in turn increases the need for continuous smoking to achieve the desired effect [27,28].

Monitoring and managing nicotine addiction at the psychological level is essential to minimize the negative health effects of this habit. Effective approaches include pharmacological therapies such as nicotine replacement agents, bupropion, and varenicline, which help reduce withdrawal symptoms and decrease nicotine cravings. Nicotine replacement agents, such as nicotine gums, patches, or lozenges, deliver nicotine in controlled doses to help relieve withdrawal symptoms and gradually reduce nicotine. Bupropion and varenicline work at the neurochemical level to reduce nicotine cravings and alleviate withdrawal symptoms [27].

Additionally, behavioral therapies play a key role in the treatment of nicotine addiction. Cognitive-behavioral therapy (CBT) helps patients identify and change negative thoughts and behavioral patterns associated with smoking. CBT teaches patients skills to cope with stress and emotions that can lead to relapse. Relaxation techniques, such as meditation and deep breathing, can also help reduce anxiety and stress [28].

Social support, in the form of treatment groups and social support programs, is extremely important in treating nicotine addiction. Support groups, such as Smokers Anonymous, offer patients the opportunity to share experiences and obtain support from people in similar situations. Social support programs, such as telephone support lines, or mobile apps, can provide patients with the tools and resources they need to maintain abstinence [29].

In summary, nicotine addiction is a complex health problem that requires a multifaceted approach. Effective treatment involves a combination of pharmacological therapies, behavioral therapies, and social support, which together can help patients achieve and maintain abstinence from nicotine.

## 3. Theoretical Framework of the Most Common Uses of Tobacco Products

In this section, we delve into the various forms of tobacco product usage, focusing on classic cigarettes, e-cigarettes, and heated tobacco products (HTPs). We provide an overview of the prevalence, health impacts, and chemical composition of these products, as well as the demographic factors influencing their use. This framework sets the stage for a detailed examination of the most common tobacco products and their effects on health.

### 3.1. Classic Cigarettes

Classic cigarettes are the most common form of tobacco use [8,9,10]. Cigarette smoking is a major contributor to mortality worldwide. It is estimated that for every single smoking-related death, there are 0.8–1.1 million cigarettes smoked [23]. The majority of smoking-related deaths are associated with cancers (especially lung, oral cavity, and throat) [4,24,25], and cardiovascular (examples include coronary artery disease, aortic aneurysm) [4,26] and respiratory diseases (examples include chronic obstructive pulmonary disease, interstitial lung disease) [4,30]. Most compulsive smokers are men. The largest group of cigarette addicts are those in the 20–39 age range. Marital status, having offspring, occupation, employment relationship, job stress, and earnings can be considered as factors influencing cigarette smoking [23,31,32]. Nearly 4800 tobacco-derived chemicals have been identified in cigarette smoke, as well as 599 tobacco additives added during the cigarette manufacturing process [33]. In addition to a very large number of toxic substances, 69 carcinogens such as polycyclic aromatic hydrocarbons (PAHs), heterocyclic compounds, N-nitrosamines, aromatic amines, aldehydes, volatile hydrocarbons, other organic compounds, and inorganic substances have been detected [33,34,35].

### 3.2. E-Cigarettes

E-cigarettes are alternative, non-flammable tobacco products—their use mimics classic tobacco smoking but without burning it [36,37]. In 2017 alone, 6.9 million people smoked e-cigarettes. Currently, there is a dramatic increase in the number of e-cigarette users, especially among young people. In 2018, the increase in e-cigarette users among 10th and 12th grade students was the largest for any substance in 44 years [37]. Unfortunately, e-cigarettes have been released without thorough preclinical toxicological testing [37]. There are more than 31 chemicals in the aerosol produced by e-cigarettes, which include nicotine, nicotirine, formaldehyde, acetaldehyde, glycidol, acrolein, acetol, and diacetyl. Glycidol is a probable carcinogen and acrolein is a potent irritant [36,38,39,40]. Smoking e-cigarettes was initially postulated as a healthy alternative to classic cigarettes; however, their unequivocal effects on human health have not been clearly established. According to some studies, their use may be associated with the development of cardiovascular [41] and respiratory [36] diseases, and possibly cancer [42].

### 3.3. Heated Tobacco Products (HTPs)

Heated tobacco products (HTPs) were launched in 2014, allowing users to inhale nicotine by heating tobacco (350 °C) instead of burning it at high temperatures [9,43]. HTPs are increasingly used, especially by adolescents and current and former smokers [10,44]. Like e-cigarettes, they have been postulated as a less harmful alternative to classic cigarettes. Although HTP smoke contains lower amounts of carbon monoxide, hydrogen cyanide, ammonia, phenol, volatile organic compounds, polycyclic aromatic hydrocarbons, aromatic amines, and reactive oxygen species than in cigarette smoke [45,46], both types of smoke contain equal amounts of tar [47]. In addition, HTP smoke contains the toxic cyanohydrin formaldehyde and high concentrations of acenapten, with potential carcinogenic properties [48,49]. Although studies show that HTP users have a lower risk of respiratory and cardiovascular diseases and cancer compared to traditional cigarette smokers, the unequivocal impact of these products on human health is not known [43,50].

## 4. Changes in Selected Biomarkers of Exposure in Users of Classic Cigarettes, E-Cigarettes, and Heated Tobacco Products—Results

The study of biomarkers of exposure in users of different tobacco products is crucial for understanding the health impacts of these products. Biomarkers can provide insights into the biological effects of tobacco use and help identify potential risks associated with different types of tobacco products [42]. By comparing the levels of specific biomarkers in users of classic cigarettes, e-cigarettes, and heated tobacco products, researchers can assess the relative harm of these products and inform public health policies [43].

Below, we provide a detailed review of the findings and discussions related to the selected biomarkers of exposure in users of classic cigarettes, e-cigarettes, and heated tobacco products.

### 4.1. Changes in the Profile of Selected Pro-Inflammatory Cytokines in Tobacco Users

Pro-inflammatory cytokines are proteins that play a key role in regulating the body’s inflammatory response. This chronic inflammation can contribute to the development of various diseases, such as chronic obstructive pulmonary disease (COPD) and cardiovascular disease [51]. In addition, chronic inflammation caused by these cytokines can lead to tissue damage and fibrosis. Understanding the role of pro-inflammatory cytokines in smokers is essential for developing targeted therapies to mitigate the deleterious effects of smoking on human health [52].

#### 4.1.1. Tumor Necrosis Factor Alpha (TNF-α)

TNF-α belongs to inflammatory cytokines and is produced mainly by macrophages and monocytes. Biologically, this cytokine is responsible for signaling phenomena in cells, leading to apoptosis or necrosis. TNF-α also mediates inflammatory processes and is responsible for cytotoxic effects [51,52].

Data on changes in TNF-α concentrations have been conducted in different types of biological material—plasma, serum, or saliva. Data on TNF-α concentrations in classical cigarette smokers are mutually exclusive. According to studies by Petrescu et al. [53], Knie et al. [54], and Barbieri et al. [55], serum TNF-α concentrations in smokers are higher than in non-smokers. Similar observations were found by Shireen et al. [56] and Tanni et al. [57] for plasma. In addition, serum TNF-α concentrations were higher in smokers who smoked more than one pack a day compared to those who smoked less than one pack a day [53]. On the other hand, some studies have shown that serum [58,59] and plasma [60] TNF- α concentrations between smokers and non-smokers do not differ. In addition, serum TNF-α concentrations did not change from baseline (interval 4 months) in subjects who underwent smoking cessation therapy [61]. As for saliva-based studies, a single study indicates that smokers have higher TNF-α concentrations, but this is not a statistically significant difference [62], while a study by Zieba et al. [63] found no difference the in levels of this cytokine in smokers and non-smokers.

For e-cigarettes and HTPs, the data on TNF-α concentrations are limited. A single study by Belkin et al. [64] indicates an increase in serum TNF-α concentrations observed up to 60 min after e-cigarette lighting. Data on potential changes at a further time interval are unfortunately not available. When tested in saliva, higher TNF-α concentrations are found in e-cigarette smokers compared to non-smokers [63,64,65,66]. Importantly, TNF-α concentrations are correlated with some periodontitis parameters [66].

In HTP users, there are no differences in serum TNF-α concentrations compared to non-HTP users [67]; however, a single study indicates that users of this form of tobacco have higher salivary concentrations of this cytokine [63]. Changes in TNF-α concentrations are shown in Table 1.

#### 4.1.2. Interleukin 1β (IL-1β)

IL-1β is a homolog of IL-1; this compound is mainly produced by macrophages, monocytes, and dendritic cells. The most important biological effects of IL-1β, in addition to mediating the inflammatory response, include a number of cellular phenomena such as proliferation, differentiation, and apoptosis [68].

As reported by Daloee et al. [58], Wang et al. [59], and Han et al. [69], the serum concentrations of IL-1β in smokers did not differ from the serum concentrations of this cytokine in non-smokers. This disagrees with the results of Barbieri et al. [55], who found higher serum IL-1β concentrations in smokers, and Shiels et al. [70], who found lower IL-1β concentrations in smokers compared to non-smokers. For determinations made in plasma, higher IL-1β concentrations are found in smokers [71,72]. Also, for saliva determinations, the data are conflicting. According to studies by Mokeem et al. [73], Kamal et al. [74], and Suzuki et al. [75], smokers have higher salivary concentrations of this cytokine compared to non-smokers. However, according to studies by Zieba et al. [63] and Alhumaidan et al. [76], there are no differences in IL-1β concentrations between the smoking and non-smoking groups. A single study using urine by Farrell et al. [77] also found no differences in IL-1β concentrations between smokers and non-smokers.

In the case of e-cigarettes and HTPs, the available data are limited and contradictory. According to the work of Singh et al. [78], higher concentrations of IL-1β are found in the plasma of e-cigarette users. Currently, there are no studies using serum. Higher concentrations of IL-1β are found in the saliva of e-cigarette smokers [73,74,78]; however, some data also indicate that IL-1β concentrations are lower in the saliva of e-cigarette smokers [63] or that there are no differences in concentrations of this cytokine between the smoking and non-smoking groups [79,80]. A single study by Farrell et al. [77] also found no differences in IL-1β concentrations in the urine of e-cigarette smokers compared to non-smokers.

In the case of HTPs, there are no studies of IL-1β concentrations in saliva, but one study found that in the serum of tobacco users, concentrations of this cytokine did not differ from those of non-smokers [67]. Changes in IL-1β concentrations are shown in Table 1.

#### 4.1.3. Interleukin 6 (IL-6)

IL-6 belongs to cytokines with multidirectional effects. It is produced mainly by monocytes and macrophages and its biological functions are related to lymphocyte activation, the induction of inflammatory processes, angiogenesis, and the induction of acute phase protein production and fever [56,74].

Most studies on the serum of smokers find elevated IL-6 concentrations compared to non-smokers. However, some research teams report that serum IL-6 concentrations do not differ between study groups [54,79,80,81,82,83]. As for plasma studies, all available literature data indicate that higher IL-6 concentrations are found in cigarette smokers compared to non-smokers [56,71,72]. However, Shireen et al. [56] found no correlation between IL-6 concentrations and smoking intensity. In the case of saliva testing, again, most studies found no differences in IL-6 concentrations between smokers and non-smokers [75,78,79,80] and only one study found higher concentrations of this cytokine in smokers [73]. A single urine study showed higher IL-6 concentrations in smokers compared to non-smokers [77].

In e-cigarette users, regardless of the material studied, elevated IL-6 values are found in smokers compared to non-smokers. Higher concentrations of Il-6 are found in the plasma [78], saliva [73], and urine [77] of e-cigarette users.

Elevated IL-6 concentrations are not found in the plasma of HTP users [81]; however, a study by Belkin et al. [75] found that IL-6 concentrations in the serum of HTP smokers are elevated up to 120 min after smoking. This may indicate that the increase in blood IL-6 concentrations is temporary. Currently, no studies are available on other body fluids of HTP users. Changes in IL-6 concentrations are shown in Table 1.

#### 4.1.4. Interleukin 8 (IL-8)

IL-8 is a potent chemotactic factor of neutrophils. This compound is produced by various cell types such as neutrophils, fibroblasts, epithelial cells, hepatocytes, macrophages, and endothelial cells. IL-8 is involved in physiological processes, but its prolonged activity is associated with a number of pathological phenomena. IL-8 is associated with the chemotaxis of neutrophils, monocytes, T lymphocytes, the promotion of inflammation and angiogenesis [84,85].

When it comes to studies conducted on the serum of classic cigarette smokers, the data are conflicting. According to some papers, smokers have higher serum IL-8 levels compared to non-smokers [54,86,87,88]. However, on the other hand, some teams have shown no difference in serum IL-8 concentrations between smokers and non-smokers [59]. A single study conducted on plasma also showed no differences in IL-8 concentrations between groups [72]. Reports on saliva are also inconsistent. As indicated by Sahibzada et al. [89] and Amirthalingam et al. [90], cigarette smokers had higher salivary Il-8 concentrations compared to non-smokers. The opposite data are provided by Frasheri et al. [91] and Karaaslan et al. [92], who both found lower IL-8 concentrations in the saliva of smokers. One study provided by Zieba et al. [63] found no differences in salivary IL-8 concentrations between smokers and non-smokers. A urine study did not show elevated IL-8 concentrations in cigarette smokers [77].

Currently, there are no studies on changes in serum IL-8 concentrations in e-cigarette smokers. However, as reported by Singh et al. [78], users of this type of tobacco have higher plasma IL-8 concentrations compared to non-smokers. Saliva data are mutually exclusive. According to Zieba et al. [63], e-cigarette smokers have lower salivary IL-8 concentrations compared to non-smokers, while a study by Karaaslan et al. [92] showed the opposite observation.

For HTPs, we have only two source reports. A serum study indicated higher IL-8 concentrations in HTP users; in addition, the concentrations increased with intake [67]. In contrast, a saliva study showed that HTP users had lower IL-8 concentrations compared to non-HTP users [63]. Changes in IL-8 concentrations are shown in Table 1.

#### 4.1.5. Interleukin 17 (IL-17)

Interleukin 17 (IL-17) is produced mainly by activated T lymphocytes. IL-17 activity is particularly important in mucous membranes, where this mediator is responsible for antimicrobial responses. IL-17 has been shown to stimulate other cells to produce inflammatory mediators such as IL-6, IL-8, and TNF-α. IL-13 is also involved in the pathogenesis of autoimmune diseases and cancer [92,93].

It is unfortunate that data on changes in IL-17 concentrations in the body fluids of tobacco users are very limited and only apply to classic cigarette smokers. A serum study by the team of Ponce-Gallegos et al. [94] showed that smokers have higher concentrations of IL-17 than non-smokers. Most data on saliva testing indicate that IL-17 concentrations in smokers and non-smokers do not differ [80,94]; however, one study indicates that smokers have higher salivary IL-17 concentrations compared to non-smokers [95]. Changes in IL-17 concentrations are shown in Table 1.

#### 4.1.6. Interferon Gamma (IFN-γ)

IFN-γ is the only interferon belonging to group II interferons. This compound is the most potent of all inflammatory mediators, activating macrophages. In addition, IFN-γ regulates apoptosis and cell proliferation, promotes antiviral activity, and also maintains the balance between Th1/Th2 lymphocytes [96,97].

Data on IFN-γ concentrations in the blood of classic cigarette smokers vary, depending on the material studied. According to the study of Daloee et al. [58], IFN-γ concentrations do not differ in the serum of smokers and non-smokers. However, in the case of plasma, higher concentrations of this mediator are found in smokers [98]. In the case of saliva studies, some studies indicate that smokers have higher IFN-γ concentrations than non-smokers [62,99]. On the other hand, a single report indicates that there are no differences in salivary IFN-γ concentrations in smokers and non-smokers [79]. One study by Farrell et al. [76] found higher urinary IFN-γ concentrations in smokers compared to non-smokers.

Higher concentrations of IFN-γ are found in the plasma of e-cigarette users than in non-smokers [77]. Higher concentrations of this mediator are also found in the urine of e-cigarette users compared to non-smokers [76]. For saliva, one study found that IFN-γ concentrations do not differ between non-smokers and e-cigarette users [62].

Only one available study looks at IFN-γ and HTPs, with no differences in salivary IFN-γ concentrations in users of this form of tobacco and non-users of HTPs [51]. Changes in IFN-γ concentrations are shown in Table 1.

**Table 1:** Changes in pro-inflammatory cytokine concentrations in users of various forms of tobacco compared to non-smokers. Meaning of the arrows: upward arrow (↑)—increase, downward arrow (↓)—decrease, and horizontal arrow (↔)—no changes.

Pro-Inflammatory Cytokines—Concentrations in Tobacco Users Compared to Non-Smokers		
TNF-α		
Classic cigarettes Serum: ↑ or ↔ [53,54,55,58,59] ↑ serum concentrations in heavier smokers [64]. Plasma: ↑ or ↔ [56,57,60] Saliva: ↔ [62,63].	E-cigarettes ↑ serum concentration up to 60 min after firing [64] Saliva: ↑ [63,65,66]	HTP Serum: ↔ serum concentrations [67]. Saliva: ↑ [63]
IL-1β		
Classic cigarettes Serum: ↓; ↑; ↔ [55,58,59,69,70] Plasma: ↑ [71,72] Saliva: ↑ or ↔ [63,73,74,75,76] Urine: ↔ [77]	E-cigarettes Plasma: ↑ [78] Saliva: ↓; ↑; ↔ [63,73,74,78,79,80]. Urine: ↔ [77]	HTP Serum: ↔ [67]
IL-6		
Classic cigarettes Serum: ↑ or ↔ [54,81,83] Plasma: ↑ [56,71,72] Saliva: ↑ or ↔ [73,75,78,79,80] Urine: ↑ [77]	E-cigarettes Plasma: ↑ [78] Saliva: ↑ [73] Urine: ↑ [77]	HTP Plasma: ↑ up to 120 min after firing [64] then ↔ [81]
IL-8		
Classic cigarettes Serum: ↑ or ↔ [54,59,86,87] Plasma: ↔ [72] Saliva: ↓; ↑; ↔ [63,88,89,90,91] Urine: ↔ [77]	E-cigarettes Plasma: ↑ [78] Saliva: ↑ or ↓ [63,91]	HTP Serum: ↑ [67] Saliva: ↓ [63]
IL-17		
Classic cigarettes Serum: ↑ [93] Saliva: ↑ or ↔ [80,94,95]	E-cigarettes No data available	HTP No data available
IFN-γ		
Classic cigarettes Serum: ↔ [57] Plasma: ↑ [98] Saliva: ↑ or ↔ [62,79,99] Urine: ↑ [76]	E-cigarettes Plasma: ↑ [77] Saliva: ↔ [62]	HTP Saliva: ↔ [62]

As can be seen, classic cigarettes show differential effects on TNF-α, IL-1β, IL-6, IL-8, IL-17, and IFN-γ concentrations. The increase in concentrations of these cytokines suggests that smoking classic cigarettes may lead to chronic inflammation, which is consistent with previous studies showing a link between smoking and inflammatory diseases. E-cigarettes also affect concentrations of pro-inflammatory cytokines, although to a lesser extent than classic cigarettes. HTPs have the least effect on concentrations of these cytokines, which may suggest that they are less harmful in the context of inflammation.

### 4.2. Changes in the Profile of Selected Anti-Inflammatory Cytokines in Tobacco Users

Anti-inflammatory cytokines are proteins that play a crucial role in regulating the body’s immune response by reducing inflammation. These cytokines counteract the effects of pro-inflammatory cytokines, preventing excessive inflammation that can lead to tissue damage [100,101,102]. Understanding the role of anti-inflammatory cytokines is essential for reducing chronic inflammation and improving overall lung health, potentially decreasing the risk of diseases such as chronic obstructive pulmonary disease (COPD), lung cancer, and cardiovascular diseases [71].

#### 4.2.1. Interleukin 4 (IL-4)

Interleukin 4 (IL-4) is produced by basophils, mast cells, and lymphocytes. The most important role of IL-4 is to antagonize the biological effects of IFN-y [100].

Higher concentrations of IL-4 are observed in the plasma of traditional cigarette smokers compared to non-smokers [101,102]. However, a study by Elisia et al. [72] conducted on plasma, and a study by Daloee et al. [58] performed on serum, found no differences in IL-4 concentrations between smokers and non-smokers. In the case of traditional smokers, higher concentrations of this interleukin are found in their saliva compared to non-smokers [79].

In the case of e-cigarettes, the scientific data are very limited. As reported by Pushalkar et al. [65], plasma IL-4 concentrations in e-cigarette users did not differ from IL-4 concentrations in non-users. It is unfortunate that there are currently no studies on IL-4 concentrations in the urine and body fluids of HTP users. Changes in IL-4 concentrations are shown in Table 2.

#### 4.2.2. Interleukin 10 (IL-10)

Interleukin 10 (IL-10) is among the cytokines with anti-inflammatory effects. This cytokine is mainly produced by macrophages, dendritic cells, B lymphocytes, and regulatory T cells [103]. IL-10 was initially referred to as a “cytokine synthesis inhibitory factor” due to IL-10’s property of inhibiting the synthesis of other inflammatory cytokines such as IFN-γ, TNF-α, IL-2, and IL-3 [53,54].

According to most scientific reports, classic cigarette smokers had lower serum IL-10 concentrations compared to non-smokers [58,59]. However, according to a study by Knie et al. [54], smokers had higher serum IL-10 concentrations than non-smokers. When assays were performed in plasma, they showed lower IL-10 concentrations in smokers compared to non-smokers [56]. Data on IL-10 concentrations in saliva and urine are poor. A study by Suzuki et al. [62] found that smokers had lower IL-10 concentrations in saliva, while urine tests showed higher IL-10 concentrations in smokers [77].

According to most studies, IL-10 concentrations in most body fluids of e-cigarette smokers are higher compared to smokers. Elevated IL-10 concentrations are found in the serum [53,54], plasma [56,57], and saliva [62,63] of e-cigarette smokers. For urine studies, no differences in IL-10 concentrations are found between e-cigarette smokers and non-smokers [66]; however, a single urine study by Singh et al. [78], found that e-cigarette smokers have lower IL-10 concentrations than non-smokers.

There are few studies on changes in IL-10 concentrations in HTP users. Both serum and plasma levels of IL-10 in HTP users are found to be higher compared to non-HTP users [67,68]. It should be noted, however, that the study by Swiatkowska et al. [67] found no differences in serum IL-10 concentrations between the two study groups (HTP users, non-users). To date, only one study has been conducted on the saliva of HTP users, where higher concentrations of IL-10 were found in users of this form of tobacco [71]. Changes in IL-10 concentrations are shown in Table 2.

#### 4.2.3. Interleukin 13 (IL-13)

IL-13 is a cytokine produced mainly by Th2 lymphocytes, eosinophils, and NK cells. IL-13 functionally and structurally resembles IL-4. This mediator is involved in fibrogenesis and parasite-fighting reactions and mediates allergic reactions. IL-13 has also been shown to play a role in cellular phenomena associated with eosinophils—survival, activation, and recruitment [104].

IL-13 concentrations in the serum of classic cigarette smokers are higher compared to non-smokers [58,78]. It is unfortunate that no data currently exist for plasma studies. As for saliva studies, the data are inconsistent. On the one hand, the teams of Farrel et al. [77] and Rodríguez-Rabassa et al. [80] found higher concentrations of IL-13 in the saliva of cigarette smokers. On the other hand, a study by Zieba et al. [63] found no statistically significant differences in IL-13 concentrations in the saliva of smokers and non-smokers. In the case of urine tests, two independent research teams found higher IL-13 concentrations in cigarette smokers compared to non-smokers [57,77].

Serum IL-13 concentrations in e-cigarette users are contradictory. On the one hand, as reported by Verma et al. [79], e-cigarette users have lower Il-13 concentrations compared to non-smokers. On the other hand, a study by Farrell et al. [77] found no differences in IL-13 concentrations between non-smokers and E-cigarette users. Plasma studies also showed a similar relationship [52,59]. As reported by Pushalkar et al. [65], higher concentrations of IL-13 are found in the saliva of e-cigarette users compared to non-smokers; however, most studies indicate that the concentration of this cytokine does not differ between the groups studied [55,56,63]. A single urine study also found no differences in IL-13 concentrations between e-cigarette users and non-smokers [60].

HTP users, according to a study by Swiatkowska et al. [67], have higher serum Il-13 concentration. Studies by Roeb et al. demonstrated no changes in serum levels among HTP users [105], but it should be noted that a study by Upadhyay et al. [106], showed that serum IL-13 concentrations of HTP users and non-users did not differ. Importantly, plasma studies also showed no differences in IL-13 concentrations between the two study groups [52,59]. Saliva studies of HTP users have shown that consumers of this form of tobacco and non-consumers of HTP have the same concentrations of IL-13 [55,56,63]. On the other hand, however, studies by Kamal et al. [74] and Farrell et al. [77] indicated that HTP consumers have higher concentrations of IL-13 in saliva compared to HTP non-consumers. One study conducted on urine showed no difference in IL-13 concentrations in HTP consumers and non-consumers [60]. Changes in IL-13 concentrations are shown in Table 2.

**Table 2:** Anti-inflammatory cytokines—concentrations in tobacco users compared to non-smokers. Meaning of the arrows: upward arrow (↑)—increase, downward arrow (↓)—decrease, and horizontal arrow (↔)—no changes.

Anti-Inflammatory Cytokines—Concentrations in Tobacco Users Compared to Non-Smokers		
IL-4		
Classic cigarettes Serum: ↔ [57] Plasma: ↑ or ↔ [71,101,102] Saliva: ↑ [79]	E-cigarettes Plasma: ↔ [64]	HTP No data available
IL-10		
Classic cigarettes Serum: ↑ or ↓ [54,58,59] Plasma: ↓ [56] Saliva: ↓ [62] Urine: ↑ [77]	E-cigarettes Serum: ↑ [53,54] Plasma: ↑ [56,57] Saliva: ↑ [62,63] Urine: ↓ or ↔ [66,78]	HTP Serum: ↑ or ↔ [67,68] Plasma: ↑ [68] Saliva: ↑ [71]
IL-13		
Classic cigarettes Serum: ↑ [58,78] Saliva: ↑ or ↔ [63,77,80] Urine: ↑ [57,77]	E-cigarettes Serum: ↑ or ↔ [77,79] Plasma: ↔ [52,59] Saliva: ↑ or ↔ [55,56,63,65] Urine: ↔ [60]	HTP Serum: ↑ or ↔ [67,105]. Plasma: ↔ [52,59]. Saliva: ↑ or ↔ [55,56,63,74,75] Urine: ↔ [60]

The analysis of the results shows that classic cigarettes have a differential effect on the concentrations of IL-4, IL-10, and IL-13. The increase in the concentrations of these cytokines may suggest that the body is trying to counteract the inflammation caused by smoking. E-cigarettes and HTPs also affect concentrations of anti-inflammatory cytokines, although to a lesser extent than classic cigarettes. The lack of data on the effect of HTPs on IL-4 concentrations makes it difficult to fully assess their health effects.

### 4.3. Changes in Growth Factor Concentrations in Users of Different Forms of Tobacco

Growth factors are proteins that play a crucial role in regulating cell growth, differentiation, and repair. They act as chemical signals that transmit information between cells, stimulating processes such as proliferation (cell reproduction), migration (movement of cells), and angiogenesis (formation of new blood vessels) [107,108]. Changes in growth factor concentrations can significantly impact various physiological processes. In users of different forms of tobacco, these changes can lead to altered cellular functions and contribute to the development of diseases. Understanding the variations in growth factor concentrations among tobacco users is essential for developing targeted therapies to mitigate the harmful effects of tobacco use on health [109].

#### 4.3.1. Transforming Growth Factor β (TGF-β)

TGF-β is involved in inhibiting cell proliferation, has pro-apoptotic effects, and is involved in wound healing [106].

Data on TGF-β are primarily related to classical smokers. According to most papers, plasma concentrations of this cytokine are higher in smokers compared to non-smokers [108,109]. The same observations were obtained by the team of Lin et al. [110], who used serum for the study. Additionally, in this study, TGF-β levels correlated with the number of cigarettes smoked. Schubert et al. [111] also found higher TGF-β concentrations in pregnant smokers compared to pregnant non-smokers. A single study also looked at saliva; classical smokers had higher salivary TGF-β concentrations compared to non-smokers [73].

Currently, the only study on TGF-β in e-cigarette users involves saliva. According to a study by Kamal et al. [74], users of this form of tobacco had higher salivary TGF-β concentrations compared to non-smokers. It is unfortunate that we do not have any studies on the effect of HTP on TGF-β concentrations in the body fluids of users of this form of tobacco. Changes in TGF-β concentrations are shown in Table 3.

#### 4.3.2. Vascular Endothelial Growth Factor (VEGF)

VEGF is a growth factor involved in angiogenesis and vascular formation during the fetal period. In addition, it increases blood vessel permeability [112].

Data on serum VEGF concentrations of classical smokers are mutually exclusive. As reported by Daloee et al. [58] and Yılmaz Şaştım, Ç et al. [113], no differences in VEGF concentrations were found between smokers and non-smokers. On the other hand, some reports indicate higher concentrations of VEGF in smokers [114,115] or lower concentrations of this growth factor in smokers compared to non-smokers [116]. Interestingly, one study of plasma from traditional cigarette smokers indicated lower concentrations of VEGF compared to non-smokers [117]. Subsequent studies have shown, however, that smokers have higher plasma VEGF concentrations [77,117]. In the case of saliva testing, studies have shown no differences in concentrations between smokers and non-smokers [113].

VEGF concentrations in the plasma of e-cigarette users are higher than in non-smokers [77]. Serum studies have yielded opposite results—e-cigarette users had lower VEGF concentrations [118]. A single saliva study showed no differences between VEGF concentrations in e-cigarette users and non-smokers [118]. Currently, there are no studies on changes in VEGF concentrations in HTP users. Changes in VEGF concentrations are shown in Table 3.

#### 4.3.3. Epidermal Growth Factor (EGF)

EGF is a growth factor responsible for regulating epidermal homeostasis—mainly by regulating the proliferation and differentiation of cells that make up this layer of skin [119].

Currently, the amount of research on changes in EGF concentrations in tobacco users is very limited. No differences in EGF concentrations are found in the serum of classic cigarette smokers compared to non-smokers [57]. In contrast, saliva studies show that EGF concentrations in smokers may be higher [120] or lower [121] than in non-smokers.

Only one study looks at EGF concentrations in e-cigarette users. A study by Ye et al. [121] found that users of this form of tobacco have lower EGF concentrations. There are no studies on HTP. Changes in EGF concentrations are shown in Table 3.

#### 4.3.4. Hepatocyte Growth Factor (HGF)

HGF is a growth factor responsible for cell growth and motility and is also important in morphogenesis [122].

Higher concentrations of HGF are found in the serum of smokers, but this is a single study [123,124]. In saliva studies, no differences are found in HGF concentrations in smokers and non-smokers [62,121].

Studies on e-cigarettes are also sparse. According to a study by Singh et al. [78], e-cigarette users have higher HGF concentrations compared to non-smokers. Saliva studies, on the other hand, are contradictory—according to Zieba et al. [63], there are no differences in HGF concentrations between e-cigarette users and non-smokers, but a study by Ye et al. [118] found lower HGF levels in e-cigarette users. Changes in HGF concentrations are shown in Table 3.

#### 4.3.5. Brain-Derived Neurotrophic Factor (BDNF)

Despite being produced mainly by neurons, BDNF crosses the blood–brain barrier. Physiologically, it is responsible for neuronal growth, neurogenesis, and neuroregeneration [125].

All studies on classic cigarette smokers involved serum or plasma. In the case of serum studies, conflicting reports have been obtained. Some studies indicate that smokers have higher serum BDNF concentrations than non-smokers [126,127,128]. On the other hand, some research teams have found no differences in BDNF concentrations between smokers and non-smokers [129,130]. However, plasma studies have shown that smokers have higher BDNF concentrations [131,132].

A single study of plasma from e-cigarette users showed no differences in BDNF concentrations compared to non-smokers [77]. Changes in BDSM concentrations are shown in Table 3.

**Table 3:** Changes in concentrations of selected growth factors in users of various forms of tobacco compared to non-smokers. Meaning of the arrows: upward arrow (↑)—increase, downward arrow (↓)—decrease, and horizontal arrow (↔)—no changes.

Growth Factors—Concentrations in Tobacco Users Compared to Non-Smokers		
TGF-β		
Classic cigarettes Serum: ↑ [109] Plasma: ↑ [107,108,110] Saliva: ↑ [73]	E-cigarettes Saliva: ↑ [73]	HTP No data available
VEGF		
Classic cigarettes Serum: ↓; ↑; ↔ [57,112,113,114,115] Plasma: ↑ or ↓ [77,116,117] Saliva: ↔ [112,121]	E-cigarettes Serum: ↓ [118] Plasma: ↑ [77] Saliva: ↔ [121]	HTP No data available
EGF		
Classic cigarettes Serum: ↔ [57] Saliva: ↓ or ↑ [120,121]	E-cigarettes Saliva: ↓ [121]	HTP No data available
HGF		
Classic cigarettes Serum: ↑ [123] Saliva: ↔ [62,121]	E-cigarettes Plasma: ↑ [77] Saliva: ↓ or ↔ [62,121]	HTP No data available
BDNF		
Classic cigarettes Serum: ↑ or ↔ [125,126,127,128,129] Plasma: ↑ [130,131]	E-cigarettes Plasma: ↔ [77]	HTP No data available

Classic cigarettes have differential effects on MCP-1, MMP-9, and CRP concentrations. Increased concentrations of these molecules suggest that smoking classic cigarettes may lead to chronic inflammation and tissue degradation. E-cigarettes and HTPs also affect the concentrations of these molecules, although to a lesser extent than classic cigarettes. These results underscore the need for further research into the effects of various forms of tobacco on the immune system and human health.

### 4.4. Changes in the Concentrations of Selected Biologically Active Molecules in Users of Different Forms of Tobacco

Biologically active molecules play a key role in the body’s immune response, regulating various physiological processes. These molecules are essential for maintaining homeostasis and ensuring the proper functioning of the immune system [132]. Changes in their concentration can significantly affect health, leading to conditions such as inflammation, tissue damage, and impaired immune response. In users of various forms of tobacco, the concentration of these biologically active molecules can change, causing cellular dysfunction and contributing to the development of chronic obstructive pulmonary disease and cardiovascular disease. Understanding these changes is essential to developing effective strategies to protect the health of tobacco users [133,134].

#### 4.4.1. Monocyte Chemoattractant Protein 1 (MCP-1)

MCP-1 is a chemokine responsible for cellular phenomena such as the recruitment of monocytes, dendritic cells, and T lymphocytes [133].

Studies of the serum of classic cigarette smokers reveal conflicting results. Some studies indicate that classic cigarette smokers have higher serum MCP-1 concentrations [134]. However, as reported by Xiromerisiou et al. [135], there are no differences in serum MCP-1 concentrations between smokers and non-smokers. In addition, Daloee et al. [58] found that lower MCP-1 concentrations are present in the serum of female smokers compared to non-smokers. For male smokers, no differences are found in the concentrations of this chemokine between the study groups. A single study was conducted using plasma, where higher MCP-1 concentrations were found in smokers compared to non-smokers [136]. Also, a single study of the saliva of classic cigarette smokers found lower concentrations of MCP-1 compared to saliva of non-smokers [137].

Studies of e-cigarette users have looked at plasma and saliva. Plasma studies have shown that MCP-1 concentrations in e-cigarette users and non-smokers do not differ [77,138]. In contrast, salivary concentrations of this chemokine are lower in e-cigarette users [62].

Currently, there is one study on HTPs, and it involves saliva, where, as with e-cigarettes, lower MCP-1 concentrations were found in HTP users than in non-users [62]. Changes in MCP-1 concentrations are shown in Table 4.

#### 4.4.2. Matrix Metalloproteinase 9 (MMP-9)

Matrix Metalloproteinase 9 (MMP-9) is an enzyme of the metalloproteinase group. Its overarching function is related to the degradation of extracellular matrix, other proteins, receptors, and antimicrobial factors [139].

MMP-9 concentrations in the serum of classic cigarette smokers are higher compared to non-smokers [140,141,142]. Importantly, MMP-9 concentrations increased with smoking duration [142]. Similar results were obtained for serum, where higher MMP-9 concentrations were also found in smokers; additionally, smokers with more than 20 years of smoking had higher MMP-9 concentrations than those with 10 years of smoking [143]. One study also looked at smokers’ saliva, which showed higher MMP-9 concentrations compared to non-smokers [144].

A study by Singh et al. [77] also found that e-cigarette users have higher plasma MMP-9 concentrations than non-users. A single urine study showed no difference in MMP-9 concentrations between the two groups [77]. Currently, we do not have any studies on HTPs. Changes in MMP-9 concentrations are shown in Table 4.

#### 4.4.3. C-Reactive Protein

CRP belongs to the acute phase proteins. It is produced in the liver and released into the bloodstream in the course of infections, trauma, cancer, and other conditions. CRP can be determined by classical or ultrasensitive methods (hs-CRP) [145].

Elevated CRP concentrations are found in the serum of classic cigarette smokers compared to non-smokers [146,147,148,149,150,151,152]. Higher concentrations are also found in ultra-sensitive CRP (hs-CRP) determinations [153]. Serum CRP concentrations did not depend on the length of smoking [149]; however, a gradual decrease in concentrations of this protein was found in those who quit smoking [151]. Also, elevated concentrations of CRP, determined by the usual [154,155] and high-sensitivity [156] methods, were found in the plasma of smokers. In the case of hs-CRP determinations, concentrations of this marker were dependent on the length of smoking time [157]. CRP concentrations in the saliva of classic cigarette smokers were also higher compared to non-smokers [78,157], while a single urine test showed no differences between smokers and non-smokers [76].

The study of CRP in the serum of E-cigarette users depends on the assay method—when classical CRP methods are used to determine concentrations between E-cigarette users and non-smokers, there are no differences in the concentrations of this protein [153]. In contrast, CRP assays using a high-sensitivity method have shown that e-cigarette smokers have higher hs-CRP concentrations compared to non-smokers [158]. Saliva testing showed higher CRP concentrations in e-cigarette users [78].

Blood tests of HTP users showed no differences in serum [66,159] and plasma [159] CRP concentrations compared to non-users. Changes in CRP concentrations are shown in Table 4.

**Table 4:** Changes in concentrations of selected biological active molecules in users of various forms of tobacco compared to non-smokers. Meaning of the arrows: upward arrow (↑)—increase, downward arrow (↓)—decrease, and horizontal arrow (↔)—no changes.

Selected Biologically Active Molecules—Concentrations in Tobacco Users Compared to Non-Smokers		
MCP-1		
Classical cigarettes Serum: ↓; ↑; ↔ [57,133,134] Plasma: ↑ [135] Saliva: ↓ [136]	E-cigarettes Plasma: ↔ [77,137] Saliva: ↓ [62]	HTP Saliva: ↓ [62]
MMP-9		
Classical cigarettes Serum: ↑ [139,140,141] Plasma: ↑ [142] Saliva: ↑ [143]	E-cigarettes Plasma: ↑ [77] Urine: ↔ [77]	HTP No data available
CRP		
Classical cigarettes Serum: ↑ [145,146,147,148,149,150,151,152] Plasma: ↑ [153,154,155] Saliva: ↑ [78,156] Urine: ↔ [76]	E-cigarettes Serum: ↑ or ↔ [152,157] Saliva: ↑ [78]	HTP Serum: ↔ [66,158] Plasma: ↔ [159]

Biologically active molecules: classic cigarettes have differential effects on MCP-1, MMP-9, and CRP concentrations. Increased concentrations of these molecules suggest that smoking classic cigarettes may lead to chronic inflammation and tissue degradation. E-cigarettes and HTP also affect the concentrations of these molecules, although to a lesser extent than classic cigarettes. These findings underscore the need for further research into the effects of various forms of tobacco on the immune system and human health.

### 4.5. Changes in the Concentrations of Selected Parameters of Oxidative Stress Parameters in Users of Different Forms of Tobacco

Oxidative stress refers to an imbalance between the production of reactive oxygen species (ROS) and the body’s ability to neutralize them with antioxidants. This imbalance can lead to cellular damage, inflammation, and impaired immune function. In users of different forms of tobacco, the concentrations of oxidative stress molecules can be significantly altered [160,161,162]. These changes can exacerbate inflammation and tissue damage, contributing to the development of diseases such as chronic obstructive pulmonary disease (COPD) and cardiovascular diseases. The accurate interpretation of these changes is crucial for developing smoking cessation and reduction programs to mitigate the health losses associated with tobacco use [163,164].

#### 4.5.1. Uric Acid

Uric acid is a product of nitrogen metabolism; in humans, it is the end product of purine base metabolism. Uric acid concentration can increase in the course of kidney disease or in gout. Uric acid comprises nearly 50% of the blood’s antioxidant capacity, but at the same time, hyperuricemia is a marker of oxidative stress [161].

Data on the serum uric acid concentrations of classic smokers are inconsistent and partly depend on the sex of the smoker. Kim et al. [162] showed that female smokers have higher elevated serum uric acid concentrations compared to female non-smokers. This study showed no such relationship for men. The study by Jang et al. [163] also showed similar observations. The studies by Murtadha et al. [164] and Hanna et al. [165] showed lower serum uric acid concentrations in smokers compared to non-smokers, regardless of gender. The opposite results were obtained by the teams of Rzoqy et al. [166] and Miguel et al. [167], who found higher uric acid concentrations in smokers of both sexes as opposed to non-smokers. Interestingly, some studies indicate that there are no differences in uric acid concentrations in the serum of smokers and non-smokers [168]. Saliva studies have shown higher uric acid concentrations in smokers compared to non-smokers [169]. However, one study found no differences in uric acid concentrations between the two groups [170]. A single urine study showed that classic cigarette smokers had higher uric acid concentrations [167]. Changes in uric acid concentrations are shown in Table 5.

#### 4.5.2. Glutathione

Glutathione is a tripeptide with antioxidant properties; it reduces thiol groups (-SH) in proteins that have been oxidized to sulfonic groups (-SO_3_H) or disulfide bonds (-S-S-). In addition, glutathione is also a coenzyme of some enzymes involved in oxidation-reduction processes [171,172].

To the best of our knowledge to date, the data on glutathione concentrations pertain only to normal smokers. Results for plasma are inconsistent. According to Bizoń et al. [173], smokers who smoke more than 20 cigarettes a day have higher glutathione concentrations, while smokers who smoke less have no differences in the concentrations of this compound. The vast majority of studies, however, indicate that no differences in glutathione concentrations are found in both the plasma and serum of smokers and non-smokers [174,175,176,177,178,179]. Currently, data on other types of biological material or other types of tobacco use are not available. Data on glutathione are presented in Table 5.

#### 4.5.3. Glutathione Peroxidase (GPx)

GPx is an antioxidant enzyme that reduces hydrogen peroxide and organic peroxides. The activity of this enzyme is dependent on the concentration of selenium ions [179].

Unfortunately, data on GPx activity in the serum of smokers are conflicting. Studies by Flohé et al. demonstrated a decrease in glutathione peroxidase levels in the serum of classic cigarette smokers [180]. According to studies by Garg et al. [181] and Ahmed et al. [182], the activity of the enzyme was higher in smokers compared to non-smokers; GPx activity also depended on the length of smoking time—higher activity was observed in smokers who smoked for longer periods of time and who smoked a greater number of cigarettes [181,182]. Higher GPx activity was also found by the team of Oladunjoye et al. [183]. However, in their study, GPx activity did not depend on the length of smoking time [183]. On the other hand, some reports indicate that cigarette smokers have lower serum GPx activity compared to non-smokers [184,185,186]. Contradictory results have also been obtained in saliva studies, with GPx activity in smokers being successively lower [187] or higher [188] compared to non-smokers. In addition, one study found no difference in salivary GPx activity between the two groups [189]. Data on GPx activity are summarized in Table 5.

To the best of our knowledge, there are currently no studies on GPx concentrations in the body fluids of e-cigarette users. However, a single study on HTP users compared to healthy subjects showed that HTP users have lower serum GPx concentrations [186].

#### 4.5.4. Superoxide Dismutase (SOD)

SOD is an enzyme of the oxidoreductase group that is responsible for inactivating superoxide anion radicals into oxygen molecules and hydrogen peroxide, which are further converted [190].

Scientific reports on SOD concentrations in classical smokers concern serum and saliva. In the case of serum, all studies indicate that smokers have lower serum SOD activity than non-smokers [181,182,184,191,192,193,194,195,196]. For saliva studies, the opposite situation was observed, as smokers had higher SOD activity compared to non-smokers [187,197,198,199].

A single saliva study of HTP users showed that they have lower SOD activity compared to non-smokers [187]. Currently, other studies involving HTP and e-cigarette users and SOD activity are not available. Data on SOD are presented in Table 5.

#### 4.5.5. Malondialdehyde

MDA is a product of lipid peroxidation and is formed by the reaction of reactive oxygen species with unsaturated fatty acids. It is a marker of oxidative stress [199,200].

MDA concentrations in the serum of classic cigarette smokers, according to studies by research teams, tend to be higher than those of non-smokers [182,183,184,192,194,201,202,203,204,205,206,207]. It should be noted, however, that one study found no difference in serum MDA concentrations between smokers and non-smokers [159]. In the case of plasma studies, MDA concentrations appear to depend on the number of cigarettes smoked per day—plasma concentrations of MDA in smokers who smoked more than 20 cigarettes per day were higher than in non-smokers [173]. Smokers smoking less than 20 cigarettes per day showed no difference in plasma MDA concentrations compared to non-smokers [175,177]. Saliva studies have clearly shown that smokers have higher MDA concentrations compared to non-smokers [63,208,209].

Reports related to MDA concentrations in the body fluids of e-cigarette smokers are severely limited. According to a study by Zięba et al. [52], smokers of this form of tobacco have higher salivary MDA concentrations. Other studies are currently not available.

Also, in HTP users, higher concentrations of MDA have been found in saliva [62]. Also, in plasma, concentrations of this marker were higher than in non-smokers [112]. Data on MDA can be found in Table 5.

#### 4.5.6. 4-Hydroxynonenal (4-HNE)

Elevated levels of 4-HNE are found in the serum of classic cigarette smokers compared to non-smokers [210,211,212,213,214,215]. Higher concentrations are also observed in the plasma of smokers [216,217,218,219,220,221,222,223,224,225], while urine tests show elevated concentrations of 4-HNE in smokers compared to non-smokers [210,211,212,213,214,215].

In the case of e-cigarette users, studies show that 4-HNE levels in serum are lower compared to classic cigarette smokers, but still higher than in non-smokers [210,211,212,213,214,215]. Plasma 4-HNE concentrations in e-cigarette users are also elevated [215,216]. Saliva tests indicate higher 4-HNE levels in e-cigarette users compared to non-smokers [220]. Urine tests reveal increased 4-HNE concentrations in e-cigarette users [211,212,213,214,215]. These changes are highlighted in Table 5.

**Table 5:** Changes in concentrations of selected parameters of oxidative stress factors in users of various forms of tobacco compared to non-smokers. Meaning of the arrows: upward arrow (↑)—increase, downward arrow (↓)—decrease, and horizontal arrow (↔)—no changes.

Selected Parameters of Oxidative Stress—Concentrations/Activity in Tobacco Users Compared to Non-Smokers		
Uric Acid		
Classic cigarettes Serum: ↓; ↑; ↔ [162,163,164,165,166,167] Saliva: ↑ or ↔ [168,169] Urine ↑ [166]	E-cigarettes Serum: ↑ [170,226] Urine: ↑ [227]	HTP No data available
Glutathione		
Classic cigarettes Plasma: ↑ or ↔ [172,173,174,176,177,178] Serum: ↔ [175]	E-cigarettes No data available	HTP No data available
Glutathione peroxidase		
Classic cigarettes Serum: ↓ or ↑ [180,181,182,183,184,185] Saliva: ↓; ↑; ↔ [186,187,188]	E-cigarettes No data available	HTP Saliva: ↓ [185]
Superoxide dismutase		
Classic cigarettes Serum: ↓ [180,181,183,190,191,192,193,194,195] Saliva: ↑ [186,196,197,198]	E-cigarettes No data available	HTP Saliva: ↓ [185]
Malondialdehyde		
Classic cigarettes Serum: ↑ or ↔ [158,181,182,183,191,193,200,201,202,203,204,205,206] Plasma: ↑ or ↔ [172,174,176] Saliva ↑: [63,207,208]	E-cigarettes Saliva: ↑ [63]	HTP Serum: ↑ [209] Saliva: ↑ [63]
4-Hydroxynonenal		
Classic cigarettes Serum: ↑ [211,212,213,214,215] Plasma: ↑ [216,217,218,219,220,221,222,223,224,225] Urine ↑ [211,212,213,214,215]	E-cigarettes Serum: ↑ [211,212,213,214,215] Plasma: ↑ [217,218,219,220,221,222,223,224,225,228] Urine ↑ [211,212,213,214,215] Saliva: ↑ [220]	HTP No data available

Classic cigarettes have varying effects on the concentrations of uric acid, glutathione, glutathione peroxidase, superoxide dismutase, malondialdehyde, and 4-hydroxynonenal. The increase in concentrations of these parameters suggests that smoking classic cigarettes may lead to increased oxidative stress, which is consistent with previous studies showing a link between smoking and oxidative stress. E-cigarettes and HTP also affect the concentrations of these parameters, although to a lesser extent than classic cigarettes.

### 4.6. Microplastic in Users of Different Forms of Tobacco

Microplastics are small fragments of plastic less than 5 mm in size that can penetrate the body and cause inflammatory reactions and the production of reactive oxygen species (ROS). Recent human studies show that microplastics can penetrate all organs, including the brain, causing behavioral changes and inflammatory reactions [229]. Similarly, studies in mice have shown that microplastic exposure leads to changes in immune markers in liver and brain tissues, indicating inflammation [230].

Exposure to microplastics in all biological systems can lead to particle toxicity, oxidative stress, inflammatory changes, and the increased absorption or translocation of these particles. The inability of the immune system to eliminate synthetic particles can result in chronic inflammation and increased cancer risk [231,232]. Studies have shown that microplastics are present in blood (both serum and plasma), saliva, and urine samples in all three groups of nicotine users. Traditional cigarette smokers showed higher concentrations of microplastics compared to e-cigarette and heated tobacco users. High microplastic concentrations were correlated with elevated levels of inflammatory biomarkers such as TNF-α, IL-1β, IL-6, IL-8, IL-17, IFN-γ, IL-10, IL-4, IL-13, TGF-β, VEGF, EGF, HGF, BDNF, MMP-9, and CRP [230].

The presence of microplastics in the bodies of nicotine users may suggest its role in the induction of inflammation. Traditional cigarette smokers are more exposed to higher concentrations of microplastics, which may be due to the fact that microplastics are found in 99% of traditional cigarette filters. Not insignificant is the presence of more toxins and chemicals present in cigarette smoke compared to alternative forms of nicotine use. Users of e-cigarettes and heated tobacco products also show the presence of microplastics, although in smaller amounts [231].

Users of various forms of tobacco who are exposed to microplastics may have an increased risk of oxidative stress. Tobacco users may be more susceptible to oxidative stress and associated cellular damage. Understanding the link between oxidative stress and microplastics is key to developing strategies to protect the health of tobacco users and reduce the risk of microplastic-related diseases [231].

## 5. Materials and Methods

This section provides a detailed description of the methodology used in this study. The article search and selection process are described, as well as the inclusion and exclusion criteria. Special attention was paid to ensuring the validity and reliability of the data analyzed.

### 5.1. Databases

Two independent authors screened titles and abstracts for relevance. Articles were searched in the following databases: PubMed, Scopus, Web of Science, and Google Scholar. The search included articles published in English and Polish.

### 5.2. Search Strategy

The search strategy consisted of the following keywords: ‘nicotine addiction’, ‘e-cigarettes’, ‘heated tobacco products’, ‘TNF-α’, ‘IL-1β’, ‘IL-6’, ‘IL-8’, ‘IL-17’, ‘IFN-γ’, ‘IL-4’, ‘IL-10’, ‘IL-13’, ‘TGF-β’, ‘VEGF’, ‘EGF’, ‘HGF’, ‘BDNF’, ‘MCP-1’, ‘MMP-9’, ‘CRP’, ‘Urid acid’, ‘glutathione’, ‘glutathione peroxidase’, ‘Superoxide dismutase’, ‘Malondialdehyde’, ‘4-HNE’, ‘microplastic’, ‘nicotine addiction therapy’, ‘blood biomarkers’, ‘saliva biomarkers’, and ‘urine biomarkers’, as well as combinations of these terms.

### 5.3. Inclusion and Exclusion Criteria

We included clinical studies, reviews, meta-analyses, and case studies regarding nicotine addiction, e-cigarettes, and heated tobacco products. We included only studies that examined biomarkers in blood, saliva, and urine in humans. We excluded all articles that described nicotine addiction in the context of social consequences such as isolation, stress, or economic problems, as well as studies using other biological materials or animal studies, in order to analyze only the direct impact of nicotine and tobacco products on the body and the development of related diseases. We specifically searched for the most recent publications to ensure the analysis was up to date. Additional inclusion criteria were studies published within the last 15 years, studies with a minimum sample size of 50 participants, and studies that provided detailed methodological information, including control conditions and statistical analyses.

### 5.4. Article Selection Process

The initial search yielded a total of 500 articles. After applying the inclusion and exclusion criteria, 150 articles were selected for further analysis. The remaining 350 articles were excluded due to not meeting the criteria.

The selected articles were evaluated based on sample sizes and control conditions to assess the reliability of the results. Detailed methodological information, such as sample sizes and control conditions, is provided in the respective sections of the selected articles.

### 5.5. Data Analysis

Relevant studies were then included with the intention of covering the widest possible spectrum of different biomarkers for nicotine addiction and related diseases. We conducted additional manual searches of the references of the related articles in order to gather information about the relevant supporting literature. The described compounds were selected after a thorough analysis of the literature. It is worth mentioning here that in the case of studies on the effects of tobacco on the body, the choice of indicators such as pro-inflammatory and anti-inflammatory cytokines, growth factors, selected biologically active molecules, selected parameters of oxidative stress, and microplastics is essential for obtaining accurate and comprehensive results.

Two independent authors screened titles and abstracts for relevance. We included clinical studies, reviews, meta-analyses, and case studies regarding nicotine addiction, e-cigarettes, and heated tobacco products. The search strategy consisted of the following keywords: ‘nicotine addiction’, ‘e-cigarettes’, ‘heated tobacco products’, ‘TNF-α’, ‘IL-1β’, ‘IL-6’, ‘IL-8’, ‘IL-17’, ‘IFN-γ’, ‘IL-4’, ‘IL-10’, ‘IL-13’, ‘TGF-β’, ‘VEGF’, ‘EGF’, ‘HGF’, ‘BDNF’, ‘MCP-1’, ‘MMP-9’, ‘CRP’, ‘Urid acid’, ‘glutathione’, ‘glutathione peroxidase’, ‘Superoxide dismutase’, ‘Malondialdehyde’, ‘4-HNE’, ‘microplastic’, ‘nicotine addiction therapy’, ‘blood biomarkers’, ‘saliva biomarkers’, and ‘urine biomarkers’, as well as combinations of these terms. We included only studies that examined biomarkers in blood, saliva, and urine in humans. We excluded all articles that described nicotine addiction in the context of social consequences such as isolation, stress, or economic problems, as well as studies using other biological materials or animal studies, in order to analyze only the direct impact of nicotine and tobacco products on the body and the development of related diseases. Relevant studies were then included, with the intention of covering the widest possible spectrum of different biomarkers for nicotine addiction and related diseases. We conducted additional manual searches of the references of the related articles in order to gather information about the relevant supporting literature.

## 6. Future Perspectives

Expanding research to include less studied forms of tobacco use, such as snus, and new nicotine delivery systems, as well as considering the potential for dual use and genetic factors, will provide a more comprehensive understanding of the health effects of tobacco. This knowledge is essential for developing effective public health strategies and improving the overall health and well-being of individuals who use tobacco products.

Nicotine Replacement Therapy (NRT) is a widely used method to help individuals quit smoking by providing a controlled dose of nicotine without the harmful substances found in tobacco smoke. NRT products include gums, sprays, patches, and inhalers. These products work by reducing withdrawal symptoms and cravings, making it easier to transition away from smoking [2,3,4]. Research has shown that NRT can significantly increase the chances of successfully quitting smoking. By providing a safer alternative to smoking, NRT helps reduce the health risks associated with tobacco use. However, it is important to use NRT products as part of a comprehensive smoking cessation program that includes behavioral support and counseling [2,3,4].

Future research should focus on optimizing NRT products to enhance their effectiveness and accessibility. Additionally, understanding the long-term effects of NRT on health and its potential interactions with other forms of tobacco use is crucial. By improving NRT strategies, we can better support individuals in their journey to quit smoking and reduce the global burden of tobacco-related diseases [2,3,4].

Snus (nicotine pouches placed under the gum) is a less studied method of nicotine intake. These products are becoming increasingly popular, especially among younger tobacco users. They have not yet been thoroughly studied, and their long-term health effects remain unclear. Studying the effects of snus on biomarkers of inflammation and other health outcomes is crucial for a full understanding of all forms of nicotine use and is essential for developing effective public health strategies [173].

Identifying specific biomarkers associated with different forms of tobacco use can lead to personalized therapeutic approaches, improving the effectiveness of smoking cessation programs [174]. Tobacco control programs, such as those described in the article published in JAMA Network Open, aim to reduce smoking prevalence and improve public health by introducing various population-level interventions [4]. Examples of such interventions include increasing taxes on tobacco products, introducing smoking bans in public places, and educational campaigns to raise awareness of the harms of smoking. By increasing knowledge about the health effects of various tobacco products on health, people can be better protected from the harmful effects of nicotine addiction [175].

Moreover, the rapid evolution of new nicotine delivery systems, such as heated tobacco products (HTPs) and e-cigarettes, necessitates continuous monitoring and research. These products are marketed as safer alternatives to traditional cigarettes, but their long-term health effects are not yet fully understood. Comprehensive studies on the impact of these products on various health biomarkers, including those related to cardiovascular, respiratory, and neurological health, are essential.

Another important area for future research is the potential for dual or poly-tobacco use, where individuals use multiple forms of tobacco products simultaneously. This behavior may have compounded health effects and complicate cessation efforts. Understanding the interactions between different tobacco products and their combined impact on health is crucial for developing effective intervention strategies.

It is also worth mentioning that microplastics may be a useful biomarker of inflammation in nicotine users. Further research is needed to better understand the mechanisms by which microplastics affect the health of nicotine users, and to assess the potential of microplastics as a biomarker of inflammation [3].

What is more, the role of genetic and epigenetic factors in individual responses to tobacco use should be explored. Personalized medicine approaches that consider genetic predispositions and epigenetic modifications could lead to more effective prevention and treatment strategies for tobacco-related diseases. By integrating genetic, environmental, and behavioral data, researchers can develop targeted interventions that address the specific needs of different populations. It is important to use NRT products as part of a comprehensive smoking cessation program that includes cognitive-behavioral support and counseling [4].

Finally, it is necessary to determine the depth of severity of addiction symptoms, motivation to stop smoking, and a multidimensional approach that also includes the assessment of tobacco use and smoking disorders, as well as the possible co-presence of other hidden or overt mental illnesses, through psychiatric and psychological assessments.

The level of nicotine addiction and motivation to stop smoking can be examined using the Modified Version of the Fagerstrom Tolerance Questionnaire (mFTQ), the Fagerström tolerance questionnaire (FTQ), the Fagerström test for nicotine dependence (FTND) [233], and the Test of Motivation for Ceasing Smoking by Nina Schneider [234].

In addition, consider using the Hamilton scale to assess mental status. The Hamilton Anxiety Rating Scale (HAM-A) is a widely used tool to assess the severity of anxiety symptoms. It consists of fourteen items, each of which assesses different aspects of anxiety, such as mood, tension, anxiety, insomnia, somatic symptoms, and others. Other mental status assessment tests that may be useful include the Beck Depression Inventory (BDI), Generalized Anxiety Disorder 7 (GAD-7), Patient Health Questionnaire-9 (PHQ-9) and the Mini-Mental State Examination (MMSE) [235].

It is also worthwhile to develop new, up-to-date tools to assess the level of nicotine dependence and motivation for cessation, covering new forms such as e-cigarettes, snus, and other new forms.

## 7. Limitations

This study has several limitations that should be acknowledged. Firstly, the availability of data on the health effects of e-cigarettes and heated tobacco products (HTPs) is limited compared to the extensive research on classic cigarettes. This disparity makes it challenging to draw definitive conclusions about the relative risks of these products. Additionally, the studies included in this review vary in their methodologies, sample sizes, and control conditions, which may introduce variability in the results.

Another limitation is the reliance on self-reported data in many of the included studies. Self-reported data can be subject to bias, as respondents may not always provide accurate information about their tobacco use habits. Furthermore, the biomarkers analyzed in this study may be influenced by factors other than tobacco use, such as diet, environmental exposures, and genetic predispositions, which were not always controlled for in the studies reviewed.

Lastly, the lack of longitudinal studies examining the long-term health effects of e-cigarettes and HTPs limits our understanding of their potential risks. Most of the available research focuses on short-term effects, and there is a need for more comprehensive studies that follow users over extended periods to better assess the chronic health impacts of these products.

Overall, these limitations highlight the need for further research with standardized methodologies, larger sample sizes, and long-term follow-ups to provide a clearer understanding of the health effects of different forms of tobacco use.

## 8. Conclusions

Nowadays, there is a growing number of people who use different forms of tobacco. While the negative impact of smoking classic cigarettes on human health has already been revealed, the exact impact of e-cigarettes and HTPs on human health is currently not clearly understood. Therefore, it is expedient to determine their exact impact on health and also to isolate from the group of tobacco users, those who are particularly vulnerable to particular disease entities. For this purpose, the use of biomarkers is advocated.

Biomarkers can help assess exposure to tobacco-specific toxicants during combustion/nicotine use, and when smokers switch from traditional cigarettes to potentially less harmful products, such as electronic nicotine delivery systems. The determination of biomarker levels will allow for the clinical monitoring of the patient’s physical condition, as all studies for diagnosing nicotinism and its level of severity are based on questionnaires and/or surveys, which respondents do not always fill out honestly.

All forms of tobacco affect the concentrations of pro-inflammatory and anti-inflammatory cytokines, growth factors, biologically active molecules, and oxidative stress parameters, which can lead to chronic inflammation and other negative health effects. Classic cigarettes seem to have the most varied impact on these biomarkers, which may result from differences in smoking habits and individual characteristics of smokers. E-cigarettes and HTPs also affect the concentrations of these biomarkers, although to a lesser extent than classic cigarettes. The lack of data on the impact of HTPs on the concentrations of some biomarkers makes it difficult to fully assess their health impact. Overall, these results highlight the need for further research on the impact of various forms of tobacco on the immune system and human health.

Measurement of inflammatory biomarkers, such TNF-α, IL-1β, IL-6, IL-8, IL-17, IFN-γ, IL-10, IL-4, Il-13, TGF-β, VEGF EGF, HGF, BDNF, MMP-9, CRP, microplastics, and selected parameters of oxidative stress, should be carried out in the context of psychiatric evaluations, taking into account the possible overlap between changes in immune and inflammatory responses in smokers, addiction disorders, and other psychiatric diseases [236]. Psychiatric disorders such as depression and anxiety are often associated with cigarette smoking, suggesting common biological mechanisms, including changes in immune and inflammatory responses [237].

Assessing selected biomarker profiles can help better differentiate these conditions, which will contribute to more effective treatment planning and intervention. In practice, assessment of exposure biomarker levels can be used to monitor the progress of treatment for patients with nicotine addiction and comorbid psychiatric disorders. Regular monitoring of these biomarkers allows assessment of the patient’s response to treatment, as well as early detection of possible relapses. For example, a decrease in TNF-α and IL-6 levels may indicate a reduction in inflammation and an improvement in the patient’s mental state, while an increase in microplastics levels may suggest an intensification of the inflammatory process. Adjusting therapy based on biomarker results allows for a more personalized approach that takes into account the individual needs of the patient, which can lead to better therapeutic outcomes.

The literature data on individual compounds are sparse and often mutually exclusive. Therefore, in order to unambiguously determine their potential, it is necessary to redo studies involving large numbers of people using various forms of tobacco.

## Abbreviations

The following abbreviations are used in this manuscript:

4-HNE	4-Hydroxy-2-Nonenal
BDNF	Brain-derived neurotrophic factor
CRP	C-Reactive protein
COPD	Chronic obstructive pulmonary disease
E-cigarettes	electronic cigarettes
EGF	Epidermal Growth Factor
et al.	et alii, and others
HGF	Hepatocyte growth factor
hs-CRP	high-sensitivity C-reactive protein
HTP	heated tobacco products
IFN-γ	Inferon gamma
IL-10	Interleukin 10
Il-13	Interleukin 13
IL-17	Interleukin 17
IL-1β	Interleukin 1β
IL-2	Interleukin 2
IL-3	Interleukin 3
IL-4	Interleukin 4
IL-6	Interleukin 6
IL-8	Interleukin 8
MCP-1	Monocyte chemoattractant protein 1
MMP-9	Matrix metalloproteinase 9
NK cell	Natural Killer cell
NRT	Nicotine Replacement Therapy
TGF-β	Transforming growth factor β
Th1 lymphocytes t	T helper type 1 lymphocytes
Th2 lymphocytes t	T helper type 2 lymphocytes
TNF-α	Tumor necrosis factor alpha
VEGF	Vascular endothelial growth factor
